# Nongenetic Optical Modulation of Pluripotent Stem Cells Derived Cardiomyocytes Function in the Red Spectral Range

**DOI:** 10.1002/advs.202304303

**Published:** 2023-11-10

**Authors:** Carlotta Ronchi, Camilla Galli, Gabriele Tullii, Camilla Marzuoli, Marta Mazzola, Marco Malferrari, Silvia Crasto, Stefania Rapino, Elisa Di Pasquale, Maria Rosa Antognazza

**Affiliations:** ^1^ Center for Nano Science and Technology Istituto Italiano di Tecnologia Milano 20133 Italy; ^2^ Humanitas Cardio Center IRCCS Humanitas Research Hospital Via Manzoni 56 Rozzano Milan 20089 Italy; ^3^ Politecnico di Milano Physics Dept. P.zza L. Da Vinci 32 Milano 20133 Italy; ^4^ Department of Chemistry, University of Bologna ‘‘Giacomo Ciamician,’’ via Francesco Selmi 2 Bologna 40126 Italy; ^5^ Institute of Genetic and Biomedical Research (IRGB) UOS of Milan—National Research Council of Italy (CNR) Milan 20138 Italy

**Keywords:** Ca^2+^ signaling, conjugated polymers, electrical activity, human pluripotent stem cell derived cardiomyocytes, optical stimulation, reactive oxygen species

## Abstract

Optical stimulation in the red/near infrared range recently gained increasing interest, as a not‐invasive tool to control cardiac cell activity and repair in disease conditions. Translation of this approach to therapy is hampered by scarce efficacy and selectivity. The use of smart biocompatible materials, capable to act as local, NIR‐sensitive interfaces with cardiac cells, may represent a valuable solution, capable to overcome these limitations. In this work, a far red‐responsive conjugated polymer, namely poly[2,1,3‐benzothiadiazole‐4,7‐diyl[4,4‐bis(2‐ethylhexyl)−4H‐cyclopenta[2,1‐b:3,4‐b’]dithiophene‐2,6‐diyl]] (PCPDTBT) is proposed for the realization of photoactive interfaces with cardiomyocytes derived from pluripotent stem cells (hPSC‐CMs). Optical excitation of the polymer turns into effective ionic and electrical modulation of hPSC‐CMs, in particular by fastening Ca^2+^ dynamics, inducing action potential shortening, accelerating the spontaneous beating frequency. The involvement in the phototransduction pathway of Sarco‐Endoplasmic Reticulum Calcium ATPase (SERCA) and Na^+^/Ca^2+^ exchanger (NCX) is proven by pharmacological assays and is correlated with physical/chemical processes occurring at the polymer surface upon photoexcitation. Very interestingly, an antiarrhythmogenic effect, unequivocally triggered by polymer photoexcitation, is also observed. Overall, red‐light excitation of conjugated polymers may represent an unprecedented opportunity for fine control of hPSC‐CMs functionality and can be considered as a perspective, noninvasive approach to treat arrhythmias.

## Graphical abstract

A red‐sensitive polymer (PCPDTBT) is proposed to modulate human pluripotent stem cell‐derived cardiomyocytes functionality. PCPDTBT photostimulation fastens Ca^2+^ dynamics, shortens action potential, accelerates beating frequency, and provides an antiarrhythmogenic effect. The interplay between photophysical transduction and biophysical pathways, involving SERCA and Na^+^/Ca^2+^ exchanger, is elucidated. This approach may offer a groundbreaking tool for noninvasive treatment of cardiac diseases.

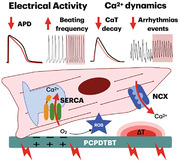

## 1 Introduction

In the last decade, optical modulation of cardiac cells and/or tissues has attracted increasing interest for the treatment of cardiovascular diseases and life‐threatening arrhythmias.^[^ ^1^ ^]^ In this field, breakthrough approaches able to provide high spatio‐temporal resolution, sensitivity, efficacy, and parallelism in both prevention and treatment, are highly desirable. Optical‐based technologies are expected to overcome typical limitations of standard pharmacological and electrical stimulation and to offer innovative solutions in cardiac repair.

To this goal, optical transduction tools that can reliably convert light into bioelectrical activity of cardiac cells are necessary. Three main options are under intensive investigation: i) use of genetically encoded light‐sensitive ion channels (optogenetics); ii) use of far red or NIR excitation, characterized by penetration depth longer than visible light (photobiomodulation, PBM); iii) use of light‐responsive nanomaterials and devices.

Optogenetic modulation demonstrated unsurpassed temporal and spatial resolution, as well as excellent selectivity and efficacy in optical control of the cardiac action potential (AP)^[^ ^2^ ^]^ and suppression of cardiac arrhythmias in transgenic animal models.^[^ ^3^ ^]^ Interestingly, optogenetic pacing has been proposed in many studies as an alternative approach to electronic pacemaker.^[^ ^2^ ^]^ However, an implantable device suitable for long‐term optical pacing has not yet been demonstrated; most importantly, issues about safety translation of optogenetic methods to human subjects have not been solved so far.

On the other side, it has been reported that PBM, a gene‐less approach based on low power (1–500 mW) delivery of photons in the far red/NIR spectrum (600–1000 nm), has a preventive and cardioprotective effect against myocardial infarction (MI), being able to partially restore the physiological state after heart failure (HF).^[^ ^4^ ^]^ This approach has been also proposed for optical pacing of cardiac cells monolayers,^[^ ^5^ ^]^ whole heart modulation,^[^ ^6^, ^7^ ^]^ and in vivo cardiac tissue control.^[^ ^8^ ^]^ Though PBM is in principle closer to translation in human subjects, unfortunately it can rarely provide the required efficacy and reliability, and it is intrinsically characterized by low spatial resolution and limited selectivity.

The third way, based on the use of optically active nanomaterials, may potentially combine the advantages of the first two ones, in terms of spatio‐temporal resolution, biocompatibility and in vivo translation capability. A few hybrid photosensitive material/cardiac cells interfaces have been recently reported, encompassing the use of both inorganic and organic materials, including graphene,^[^ ^9^ ^]^ polymer‐silicon nanowires,^[^ ^10^ ^]^ gold nanoparticles,^[^ ^11^ ^]^ organic molecules.^[^ ^12^ ^]^

The use of carbon‐based semiconductors emerged as a highly promising strategy for optically‐driven cell control, given their optimal properties in terms of in vivo long‐term implantability,^[^ ^13^ ^]^ excellent optoelectronic coupling, as well as phototransduction efficiency and reliability, as demonstrated in several cell types, such as neurons,^[^ ^14^, ^15^, ^16^, ^17^ ^]^ astrocytes,^[^ ^18^ ^]^ stem cells,^[^ ^19^, ^20^ ^]^ and endothelial cells.^[^ ^21^, ^22^, ^23^, ^24^ ^]^ Surprisingly, cell optical modulation by conjugated polymers has been very rarely addressed in the cardiovascular field. In recent works, thiophene‐based polymer thin films (poly(3‐hexylthiophene, P3HT) promoted proliferation and angiogenesis of endothelial cells^[^ ^21^ ^]^ and modulated both the contraction rate^[^ ^25^ ^]^ and the redox balance^[^ ^26^ ^]^ in cardiac cells. A critical aspect of these first proof‐of‐concept interfaces was represented by the use of a green‐light absorbing material, which represents a strong limitation to the implementation and translation of a therapeutically viable approach. Moreover, the biological pathways involved in the phototransduction process were not addressed in detail, thus limiting the possibility for a further improvement of the property of the material by rational design.

As a necessary step toward in vivo applications, with the present work we aimed at both i) developing a new photoactive interface, with optical responsivity in the far red/NIR range, while fully preserving cytocompatibility and phototransduction efficiency typical of exogenous conjugated polymers, and ii) elucidating the biophysical mechanisms underlying optical coupling between organic materials and cardiac cells.

To this purpose, hPSC‐CMs were used as a valuable and widely recognized cell model for cardiac studies.^[^ ^27^ ^]^ As the photoactive material counterpart, we employ a low bandgap conjugated polymer, namely PCPDTBT, characterized by far‐red optical absorption, excellent charge photogeneration efficiency, and optimal photoelectrochemical stability in a biological environment.

We observe a sizable modulation of the hPSC‐CMs functionality, we provide strong experimental evidence of the capability of PCPDTBT/hPSC‐CMs interfaces to optically control intracellular Ca^2+^ dynamics and electrical activity, and we identify the phototransduction pathways at play. Overall, our results fully support the development of a contactless and geneless approach, based on bio‐organic photonics, to treat arrhythmias with unprecedented spatial and temporal resolution, while enabling lower invasiveness and higher selectivity. We believe they may serve as a starting point for further engineering of the biohybrid interface for cardiac repair applications.

## 2 Results and Discussion

### 2.1 Preparation and Characterization of the Hybrid hPSC‐CMs/Polymer Interface, in Dark

PCPDTBT is a prototype conjugated polymer characterized by a low energy gap and a high charge generation efficiency.^[^ ^28^ ^]^ Due to its distinct optoelectronic properties, it has been widely employed for the realization of organic photovoltaic cells,^[^ ^29^, ^30^, ^31^ ^]^ but, to the best of our knowledge, it was only rarely considered for bioelectronics applications.^[^ ^14^, ^16^, ^32^, ^33^, ^34^, ^35^, ^36^ ^]^ Figure S1A (Supporting Information) shows the PCPDTBT optical absorption spectrum and its chemical structure.

**Figure 1:**
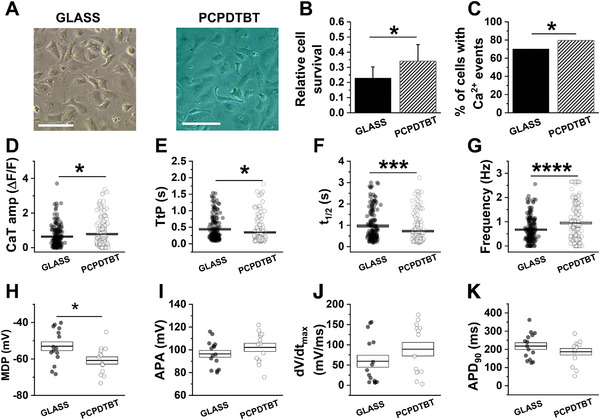
PCPDTBT modulation of hPSC‐CMs functionality in dark condition. A) images of hPSC‐CMs plated on GLASS and PCPDTBT. Scale Bar: 200 µm. B) Cell viability assessed by MTT assay. C) Quantitative analysis of the number of cells exhibiting Ca^2+^ transient. D–G) Ca^2+^ dynamics parameters: Ca^2+^ transient amplitude (CaT amp, D), Time t to peak (TtP, E), Half time Ca^2+^ transient decay (*t*_1/2_, F) and spontaneous beating frequency (Frequency, G). H–K) Electrical activity parameters: maximal diastolic potential (MDP, H); action potential amplitude (APA, I); maximal upstroke velocity (d*V*/d*t*_max_, J); action potential duration at 90% of repolarization (APD90, K). Individual and average ± SE (box) values are shown in each dot plot. Ca^2+^ measurements: GLASS, *N* > 47, PCPDTBT, *N* > 60. Electrical activity measurements: GLASS, *N* = 15, PCPDTBT, *N* = 13. * *p* < 0.05, *** *p* > 0.01, **** *p* < 0.001 GLASS versus PCPDTBT (Student's *t*‐test).

For the realization of the hybrid bio‐polymer interface, hPSC‐CMs were plated on top of the conjugated polymer, as well as on control GLASS substrates (**Figure** **1**A). No relevant morphological changes are evidenced. Since PCPDTBT was never reported as a cell culturing substrate for CMs, nor for other kinds of stem cells‐derived cells, we first evaluated its compatibility with hPSC‐CMs seeding and growth by using a standard proliferation assay, based on cellular metabolic activity detection (MTT assay). Figure 1B shows that hPSC‐CMs cell survival is not only fully maintained on PCPDTBT, but it is even significantly higher, as compared to control samples onto GLASS. This result is consistent with a previous study, where PCPDTBT successfully promoted cell viability in a model cell line (HEK‐293) after 3 days of culture, as compared to control and other conjugated polymers.^[^ ^32^ ^]^ The authors reported a PCPDTBT surface roughness of 4.5 nm root mean square value, calculated at 1 µm length scale, which slightly increased upon addition of fibronectin (by a factor of 1.1). It has been demonstrated on multiple cell types that the optimization of the surface roughness can modulate the interaction between surface materials and cells, improving cell survival.^[^ ^37^, ^38^ ^]^ In the specific case of CMs derived from embryonic stem cell, the increased roughness of poly(ethylene terephthalate)/dilinoleic acid (PET/DLA) induced by TiO_2_ has been associated to a better CMs adhesion and differentiation;^[^ ^39^ ^]^ Stout et al.^[^ ^40^ ^]^ demonstrated that nanoroughness of carbon nanofibers (CNF) embedded in poly(lactic‐*co*‐glycolic‐acid) (PLGA) increased CMs function as it mimics that of the native heart tissue. Thus, we hypothesize that the nanoroughness of the PCPDTBT surface, as already evaluated^[^ ^32^ ^]^ and expected to be the same given the unchanged fabrication protocol, might contribute as well to cell survival increment and ultimately to intrinsically modulate the functionality of hPSC‐CMs, even in absence of photoexcitation. To investigate this possibility, two key features of CMs have been evaluated in dark condition, namely i) Ca^2+^ ion dynamics (Figure 1C–G) and ii) electrical activity^[^ ^41^ ^]^ (Figure 1H–K).

Spontaneous Ca^2+^ transients were measured as fluorescence relative values (Δ*F*/*F*) of Fluo4‐AM, a dye sensitive to intracellular Ca^2+^ concentration changes, which increases its fluorescence upon Ca^2+^ binding. We employed Fluo4‐AM since its excitation/emission spectra do not overlap with the PCPDTBT optical absorption/fluorescence (Figure S1A, Supporting Information). The excitation of Fluo4‐AM was provided by a blue light emitting diode (LED), with emission centered around 474 nm and power density of 5.8 mW mm^−2^. The following parameters were evaluated for Ca^2+^ dynamics recording (Figure S2A, Supporting Information): Ca^2+^ transient (CaT) peak amplitude (CaT amp), defined as the difference between the fluorescence peak value and the baseline value, or Δ*F*/*F*; time to peak (TtP), defined as the time difference between the CaT onset and the CaT maximum; half‐time CaT decay (*t*_1/2_), defined as the half‐time decay of CaT. Spontaneous beating frequency (Frequency) was also calculated, as the number of CaT events in the time unit. Interestingly, a quantitative analysis of the percentage of hPSC‐CMs exhibiting CaTs reveals that their incidence is significantly higher (+10%) in cells cultured on PCPDTBT compared to those on GLASS (Figure 1C), thus providing further evidence of the positive outcome of polymer substrates on cells functionality. Furthermore, hPSC‐CMs plated on polymer show: higher CaT amp (Figure 1D); faster TtP and *t*_1/2_ (Figure 1E,F), and higher spontaneous CaT frequency (Figure 1G), as compared to control samples on GLASS. All these features resemble those of mature CMs,^[^ ^42^, ^43^, ^44^ ^]^ thus supporting a modulation of hPSC‐CMs biological properties and functionality by PCPDTBT thin films, even when used as culturing substrates without optical excitation. Interestingly, these data are in line with those obtained in HL‐1 cells cultured on electrically‐conductive polymeric substrates (polypyrrole‐polycaprolactone, PPy‐PCL).^[^ ^45^ ^]^

In order to determine a more comprehensive profile of the functional properties of hPSC‐CMs when plated on PCPDTBT, we assess their electrophysiological activity. Experiments were performed on hPSC‐CMs, electrically paced at 1 Hz, by using the patch clamp technique. The following parameters of the action potential (AP) signal were evaluated (Figure S2B, Supporting Information): maximum diastolic potential (MDP), defined as the rest membrane potential (*V*_m_); AP amplitude (APA), defined as the potential difference between the AP peak and the MDP value; maximum upstroke velocity (d*V*/d*t*_max_), defined as the first derivative of the AP depolarization phase; AP duration at fixed time points during the repolarization phase (APD90, APD50, APD20), defined as the time difference between the AP onset and the timing at which the AP value has decreased by 90%, 50%, 20%, respectively. hPSC‐CMs plated on PCPDTBT presented a significantly hyperpolarized MDP compared to hPSC‐CMs grown on GLASS (−61 ± 2 vs −53 ± 2 mV, *p* < 0.05, Figure 1H), whereas the APA, the d*V*/d*t*_max_ and the APD90 were not significantly different between the two groups (Figure 1I–K). The depolarized MDP is a typical feature of immature CMs;^[^ ^46^, ^47^ ^]^ fetal‐like phenotype of CMs generated from hPSCs is one of the major limitations to their use for disease modeling, drug testing, and regenerative medicine applications, and efforts are being made to overcome this issue by promoting the hyperpolarization of membrane potential,^[^ ^48^ ^]^ including approaches based on optogenetics.^[^ ^49^ ^]^

Overall, data in Figure 1 clearly indicate that PCPDTBT shifts the MDP toward values closer to those of mature CMs (Figure 1H), and acts on Ca^2+^ ion dynamics, inducing the establishment of a more mature pattern of the main functional properties of the CaT (Figure 1D–G).

### 2.2 Optical Modulation of Ca^2+^ Ion Dynamics

**Figure 2:**
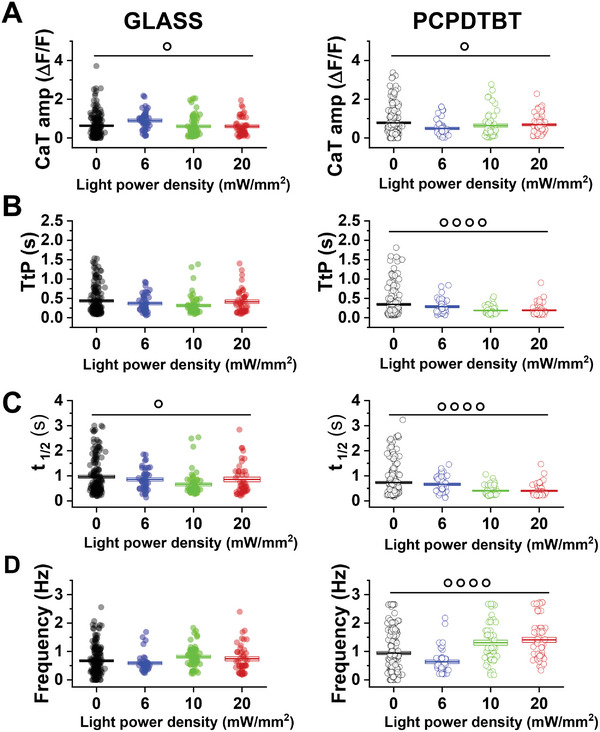
Light power density dependence of Ca^2+^ dynamics in hPSC‐CMs plated on GLASS (left) or PCPDTBT (right). A) Ca^2+^ transient amplitude (CaT). B) Time to Peak (TtP). C) half time decay (*t*_1/2_) and D) spontaneous beating frequency (Frequency). Individual and average ± SE (box) values are shown in each dot plot. GLASS and PCPDTBT, *N* > 50. °*p* < 0.05, °°°*p* < 0.001, °°°°*p* < 0.0001 within each group (ANOVA one‐way test). *p < 0.05 GLASS versus PCPDTBT (Chi‐square test).

Prior to investigate the effect of the PCPDTBT‐mediated photostimulation on hPSC‐CMs, we tuned the excitation protocol to avoid detrimental effects on cells. In fact,  stimulation of opto‐electrically active materials may lead to phototoxicity effects, in a not‐straightforward manner, depending on several variables, including the light protocol (power density, frequency, duration, wavelength), the optical absorption of the material, its optoelectronic efficiency, heat conductivity properties, and the considered cellular model. One of the most critical parameters is the light power density; thus, we preliminarily identify a safe range in the case of hPSC‐CMs cultured on glass and on PCPDTBT, by carrying out a light/dose Ca^2+^ response analysis (**Figure** **2**).

Three different power densities (6, 10, and 20 mW mm^−2^) were applied for 30 s in different batches of cells. At first, we tested hPSC‐CMs seeded on optically transparent, control substrates (GLASS), to assess the intrinsic effects of red light illumination (Figure 2, left). On GLASS, red‐light illumination significantly decreases CaT amplitude and accelerates CaT decay (*t*_1/2_), while it does not affect the TtP value nor the beating frequency. Similar to our results, Dittami et al.^[^ ^50^ ^]^ showed that the Ca^2+^ events evoked by infrared (IR) pulses (≈1862 nm) presented a reduced Ca^2+^ amplitude and faster *t*_1/2_ compared to spontaneous Ca^2+^ events. The authors also demonstrated that mitochondria contribute to the IR‐evoked Ca^2+^ events; it has been suggested that cytochrome C of the mitochondrial respiratory chain might absorb both red and IR light.^[^ ^51^, ^52^ ^]^ This evidence suggests that mitochondria may be involved in the red‐light effect on Ca^2+^ dynamics observed in our experiments, though additional experimental proof is required to confirm this hypothesis. On the contrary, Zhang et al.,^[^ ^53^ ^]^ reported that the exposure to 2.5 mW mm^−2^ of LED red‐light (630 nm) for 10 min does not change the CaT of neonatal mouse CMs compared to control cells. The discrepancy respect to our results may originate from different lighting protocols, and it could be ascribed to the longer exposure to light (e.g., increased amount of deposited energy at comparable light density).

More interestingly, PCPDTBT photoexcitation resulted in a significantly faster TtP, *t*_1/2_ decay and beating frequency (Figure 2, right). CaT amplitude decreased under illumination also in polymer group, similar to GLASS, suggesting that this effect could be unrelated to polymer photoexcitation (Figure 2A, right). Conversely, TtP, *t*_1/2_ and frequency values show a highly statistically significant variation in presence of polymer photoexcitation, as compared to dark condition (Figure 2B–D, right). We conclude that red‐light stimulation of PCPDTBT preserves the physiological functionality of hPSC‐CMs at all the investigated power densities, and it leads to sizable modulation of the Ca^2+^ homeostasis in a power‐density dependent manner, with larger changes observed at the highest tested power density.

Based on this outcome, the measurements reported in the following were carried out at 20 mW mm^−2^ and by exposing cells to red light excitation for 30 s. Importantly, we also assessed the reversibility of the light‐induced effects, by recording Ca^2+^ dynamics also after light switching off. The time protocol is shown in Figure S3 (Supporting Information).

**Figure 3:**
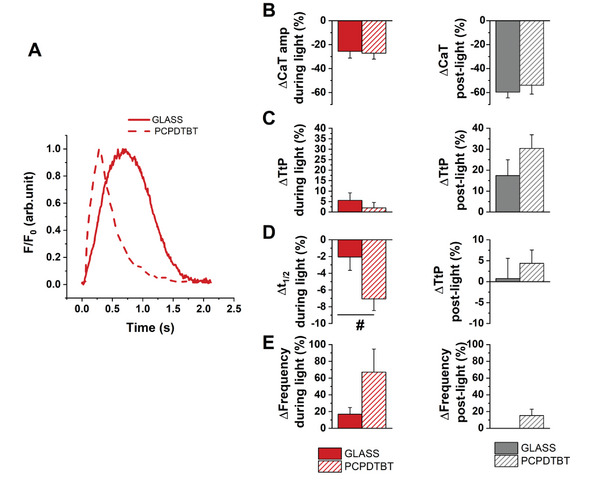
Effect of photostimulation of Ca^2+^ dynamics within the same hPSC‐CMs plated on GLASS or on PCPDTBT. A) Normalized representative traces of Ca^2+^ transient of hPSC‐CMs plated on PCPDTBT under illumination. Percentage of relative variation between dark condition versus illuminated samples (left) and post‐light conditions (right) of Calcium transient amplitude (CaT amp, B), Time t to peak (TtP, C), Half time Ca^2+^ transient decay (*t*½, D) and spontaneous beating frequency (Frequency, E). GLASS, *N* = 55, PCPDTBT, *N* = 73. #*p* < 0.05 GLASS versus PCPDTBT.

**Figure** **3** shows Ca^2+^ dynamics and relevant parameters obtained in the two cohort samples, control GLASS and photoactive PCPDTBT samples, over the very same ROIs before, during and after photoexcitation. Figure 3A compares representative CaT recorded upon photoexcitation, evidencing shorter decay time in polymer groups compared to the glass one. Ca^2+^ dynamics parameters (Figure 3B–E) are shown as percentage relative variations between dark conditions (e.g., prior to photoexcitation) versus illuminated samples (e.g., “during light,” red histograms), as well as percentage relative variations between pre‐ versus postlight conditions (e.g., “post light,” gray histograms). During photostimulation, CaT significantly lowered in both GLASS (−26% ± 5%) and PCPDTBT (−27% ± 5%) samples with respect to dark (Figure 3B left; and Figure S4A, Supporting Information). The effect did not recover after illumination, being CaT about 60% (GLASS) and 54% (PCPDTBT) lower than in the dark condition (Figure 3B right). No differences were detected between TtP values throughout all the measurements in both groups (Figure 3C; and Figure S4B, Supporting Information). Interestingly, *t*_1/2_ value significantly decreased only in photoexcited PCPDTBT as compared to dark (Figure S4C, Supporting Information) and this variation was higher respect to GLASS (−2% ± 1% vs −7% ± 1%, *p* < 0.05, Figure 3D left). This result indicates the presence of higher CaT decay time, caused by polymer photostimulation, fully recovered after illumination (about 4% higher than in the dark condition, Figure 3D right). A light‐induced increase of the beating frequency was also present in both groups (+17% ± 8% GLASS vs +67% ± 28% PCPDTBT, Figure 3E left), which resulted statistically significant only in the PCPDTBT case (Figure S4D, Supporting Information). After optical stimulation, the beating frequency show persistent increase in the PCPDTBT group (15% ± 7%), but not in the GLASS condition (Figure 3E right). These latter results are in line with a previous work,^[^ ^25^ ^]^ reporting an enhancement in the contraction rate of hPSC‐CMs upon green‐light excitation of the rr‐P3HT conjugated polymer.

### 2.3 Optical Modulation of Electrical Activity

**Figure 4:**
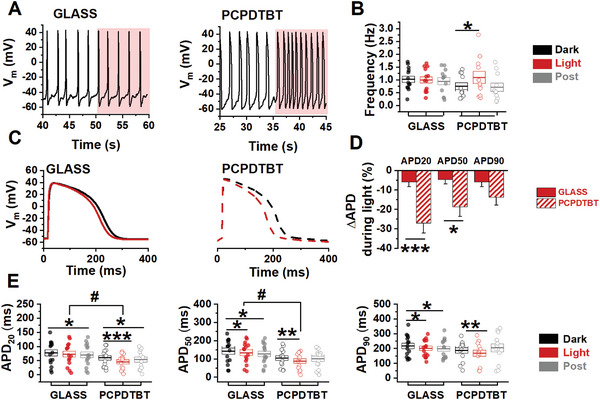
Optical stimulation of electrical activity of hPSC‐CMs. Action Potential parameters evaluated before (Dark), during (Light), and after (Post) illumination in spontaneous beating iPSC‐CMs A,B) or during a constant rate pacing at 1 Hz C–E). A) Example of spontaneous electrical activity in dark condition and under illumination (area shaded in purple). B) Spontaneous beating frequency. C) representative traces of AP of hPSC‐CMs plated on glass (left) and on PCPDTBT (right). D) Light induced effect on action potential (AP) duration normalized over dark condition values. E) AP duration (APD) at 20%, 50%, and 90% of repolarization. Individual and average ± SD (box) values are shown in each dot plot. GLASS, *N* >10, PCPDTBT, *N* >10. *p < 0.05 and **p < 0.01 dark versus light, #*p* < 0.05 GLASS versus PCPDTBT (Student's *t*‐test).

Next, the effect of PCPDTBT photoexcitation on hPSC‐CMs electrical activity was investigated. To this purpose, we recorded AP signaling by patch clamp upon the same illumination protocol used for Ca^2+^ measurements (Figure S3, Supporting Information) and we evaluated the AP parameters (see Figure S2B, Supporting Information) in hPSC‐CMs plated either on GLASS or on PCPDTBT (**Figure** **4**). In dark conditions, the spontaneous beating rate is similar in both groups. Notably, the photostimulation enhances the beating rate (+45%) only in the PCPDTBT group (Figure 4B) in accordance with Ca^2+^ recordings (Figure S4D, Supporting Information). The increment is reversible since it recovers to baseline values after the illumination (Figure 4B).

PCPDTBT polymer thin films were previously used by Feyen et al. as photoactive interfaces with hippocampal slices;^[^ ^16^ ^]^ in that case, the illumination of the polymer surface triggered a sustained hyperpolarization of the neural membrane and it significantly reduced neuronal firing. Conversely, in our case no difference in MDP was observed during optical stimulation (data not shown); thus, we infer that the increase in the beating rate observed upon photostimulation is not related to *V*_m_ depolarization.

To remove the APD rate dependence,^[^ ^54^ ^]^ the optical modulation of APs was also investigated in hPSC‐CMs electrically stimulated at 1 Hz (Figure 4C–E). In hPSC‐CMs plated on PCPDTBT, photoexcitation significantly shortens APD20, APD50, and APD90. A slight intrinsic effect of sole light condition is visible also in GLASS samples as APD50 and APD90 shortening; however, the effect in PCPDTBT samples is much higher, by about 2 times (APD90), 4 times (APD50), and 5 times (APD20) (Figure 4D). In PCPDTBT group, APD shortening reverted (toward APD prolongation) after photoillumination, although not reaching the preillumination values, showing that the APD optical modulation is partially reversible (Figure 4E). MDP hyperpolarization was maintained also during illumination of PCPDTBT; APA and d*V*/d*t*_max_ did not show relevant differences in both groups (Figure S5, Supporting Information).

Overall, these results show that PCPDTBT photoexcitation efficiently modulates the hPSC‐CMs electrical activity regardless of the beating rate, and are in line with changes observed in Ca^2+^ homeostasis (Figures 2 and 3).^[^ ^55^, ^56^ ^]^

Other photosensitive transducers were also proposed as tools to regulate the cardiac cell functions. For example, photoexcited graphene has been used to trigger membrane depolarization in CMs, allowing to generate or increase the frequency of APs.^[^ ^57^ ^]^ The employment of this nanomaterial, however, necessitates further investigation to study the bio‐toxic effects of different types of graphene. Gold nanoparticles‐treated neonatal rat CMs have shown light induced regulation of the beating frequency, caused by a thermally activated mechanism.^[^ ^58^ ^]^ Polymer–silicon nanowires have been employed to regulate the beating frequency of isolated rat CMs and hearts, by taking advantage of photogenerated faradaic currents that act similarly as in electrical pacing stimulation.^[^ ^10^ ^]^ The main limitation of silicon nanowires and gold nanoparticles‐based methods, as compared to our approach, lies in the employment of green light which presents a very low penetration within living tissues. Another alternative is represented by the exploitation of photochromic compounds, bounded covalently to an ion channel or not‐covalently to the plasma membrane. We have recently demonstrated that the photoactivation of an intramembrane photochromic transducer, Ziapin2, enhances both the frequency of Ca^2+^ transients and the contraction rate of hPSC‐CMs.^[^ ^59^ ^]^ However, the biophysical mechanism ruling the photostimulation of Ziapin2‐loaded hiPSC‐CMs is not yet fully elucidated, making a direct comparison with the present work not straightforward.

### 2.4 Optically‐Driven Activation of Physiological Pathways

A pharmacological investigation was then designed to understand the mechanisms underlying the PCPDTBT‐mediated photomodulation of hPSC‐CMs functionality. Based on the results above, we hypothesize that PCPDTBT could potentially modulate two important Ca^2+^‐handling proteins involved in the cytosolic Ca^2+^ removal during the CaT decay^[^ ^60^ ^]^: i) SERCA, which reuptakes the cytosolic Ca^2+^ into the sarcoplasmic reticulum (SR) and/or ii) NCX, which is an antiporter membrane protein that removes intracellular Ca^2+^.

**Figure 5:**
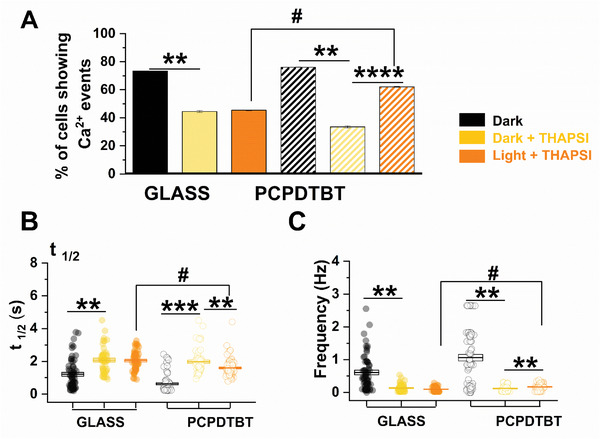
Photomodulation of Ca^2+^ decay in the presence of a SERCA blockade. iPSC‐CMs treated with 10 µm Thapsigargin (THAPSI) in dark (Dark+THAPSI) and upon photostimulation (Light+THAPSI) compared with values obtained in dark condition (Dark). A) Quantitative analysis of the number of cells exhibiting Ca^2+^ transients. B) Half time Ca^2+^ transient decay (*t*_1/2_) C) Spontaneous beating frequency (Frequency). GLASS: Dark, *N* = 55, Dark+THAPSI, *N* = 52, Light+THAPSI, *N* = 56; PCPDTBT: Dark, *N* = 73, Dark+THAPSI, *N* = 53, Light+THAPSI, *N* = 85. *p < 0.05 and ***p < 0.001 versus Dark; #*p* < 0.01 GLASS versus PCPDTBT.

To assess the contribution of SERCA, hPSC‐CMs were exposed to Thapsigargin (10 µm, THAPSI), a selective blocker^[^ ^61^ ^]^ of SERCA, for 15 min; the protocol was properly designed in order to not completely abolish the Ca^2+^ dynamics (**Figure** **5**).^[^ ^62^ ^]^ In dark condition, the application of THAPSI significantly reduced the percentage of cells showing Ca^2+^ events (−39.2% GLASS and −55.8% PCPDTBT, Figure 5A), prolonged t_1/2_ (Figure 5B) and decreased the spontaneous beating frequency in both groups (Figure 5C). The optical stimulation did not change Ca^2+^ parameters in CMs plated on GLASS. On the contrary, PCPDTBT samples treated with THAPSI show an opposite behavior under illumination, and in particular an enhancement in the number of hPSC‐CMs exhibiting CaTs (+85%), a reduction of *t*_1/2_ and an increase in the beating frequency. This suggests that PCPDTBT photoexcitation efficiently stimulates SERCA activity. A similar effect was observed with a pharmacological SERCA activator in human CMs from induced PSC derived from patients with a reduced SERCA activity^[^ ^62^ ^]^ and in rats with diabetic cardiomyopathy.^[^ ^63^ ^]^ Moreover, SERCA has been reported to be a valid target in cardiac diseases such as in heart failure and arrhythmias.^[^ ^64^ ^]^

**Figure 6:**
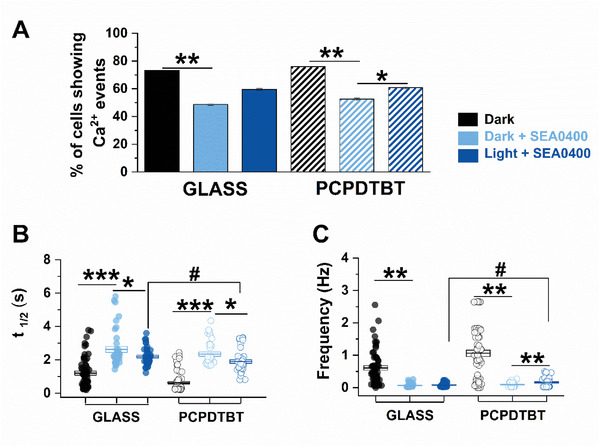
Photomodulation of Ca^2+^ decay in the presence of a Na^+^/Ca^2+^blockade. iPSC‐CMs treated with 2 µm SEA0400 (SEA) in dark (Dark+SEA) and upon photostimulation (Light+SEA). Data were compared with values obtained in dark condition (Dark). A) Quantitative analysis of the number of cells exhibiting Ca^2+^ transient. B) Half time Ca^2+^ transient decay (*t*_1/2_) C) Spontaneous beating frequency (Frequency). GLASS: Dark, *N* = 55, Dark+SEA, *N* = 39, Light+SEA, *N* = 43; PCPDTBT: Dark, *N* = 73, Dark+SEA, *N* = 34, Light+SEA, *N* = 43. *p < 0.05 and ***p < 0.001 versus Dark; #*p* < 0.01 GLASS versus PCPDTBT.

To investigate the role of NCX, CaTs were recorded in the presence of the NCX blocker SEA0400 (2 µm, **Figure** **6**).^[^ ^65^ ^]^ As expected,^[^ ^61^ ^]^ in dark condition the inhibition of NCX reduced the number of cells showing CaT, while those still exhibiting CaTs had prolonged *t*_1/2_ and decreased spontaneous beating rate, in both polymer and control samples. In the presence of SEA0400, the optical stimulation induced a slight shortening of the *t*_1/2_ similarly in both GLASS and PCPDTBT (Figure 6B). These results, together with those shown in Figure 2C, suggest that red‐light stimulation by itself, regardless of PCPDTBT mediation, influences the CaT decay, by modulating NCX activity. Noteworthy, however, under NCX blockade, the number of hPSC‐CMs exhibiting CaT was significantly higher upon illumination in PCPDTBT (Figure 6A), and these cells showed a shorter *t*_1/2_ and a faster beating rate comparing with CMs in dark condition (Figure 6B,C).

One of the mechanisms proposed to explain hPSC‐CMs spontaneous activity is a periodic Ca^2+^ cycling, named “Ca^2+^ clock”^[^ ^66^ ^]^: spontaneous Ca^2+^ releases from SR gradually enhance the cytosolic Ca^2+^ concentration which activates the NCX forward mode, resulting in an inward net current and thus a *V*_m_ depolarization potentially sufficient to trigger APs; SR refill then requires cytosolic Ca^2+^ reuptake via SERCA. Overall, our results converge to sustain that the PCPDTBT optical stimulation efficiently influences the hPSC‐CMs spontaneous beating through the stimulation of the SERCA and NCX activity. This opens the way to several different applications, wherever the possibility to modulate CMs activity in a geneless, touchless, selective, and spatio‐temporally resolved manner would be key, both for fundamental knowledge and for possible therapeutic outcomes.

### 2.5 Phototransduction Mechanisms

Data reported so far unequivocally demonstrate that PCPDTBT photoexcitation turns into optical modulation of hPSC‐CMs electrical activity and intracellular Ca^2+^ homeostasis through SERCA and NCX pathways activation. However, the phototransduction mechanisms active at the cell/polymer interface remain to be clarified at this stage. Increasing experimental evidence on other in vitro cell models demonstrated the occurrence, sometimes in parallel, of chemical/physical processes, triggered by photoexcitation and subsequent induction at the polymer surface of capacitive charging, temperature increase, faradaic reactions, and ion species imbalance in the extracellular space.^[^ ^16^, ^18^, ^22^, ^26^, ^67^ ^]^ Relevant to this study, regulation of the Ca^2+^ handling proteins by redox signaling and reactive oxygen species (ROS) generation have been widely observed in the heart, both in physiological and pathological conditions. The latter are characterized by excessive ROS production, such as in chronic cardiac remodeling leading to heart failure.^[^ ^68^, ^69^, ^70^ ^]^ In addition, our recent studies revealed that optical excitation of a green‐light absorbing P3HT film leads to ROS production and to consequent intracellular Ca^2+^ variation and angiogenic response in endothelial cells.^[^ ^21^, ^22^, ^71^ ^]^

**Figure 7:**
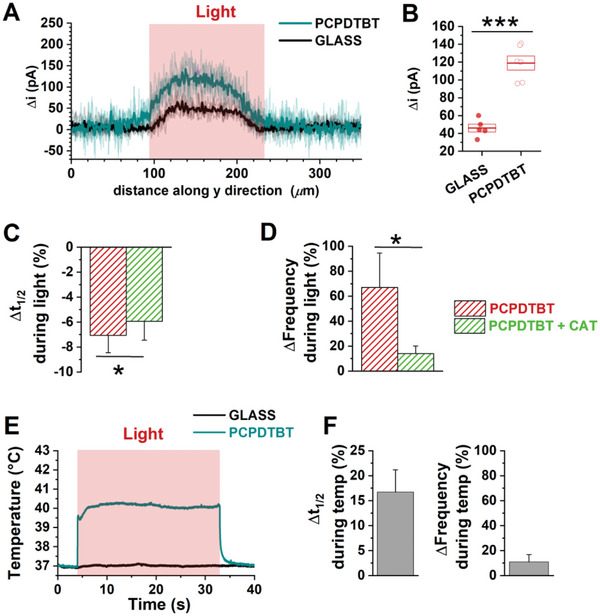
Investigation of the PCPDTBT‐mediated photo transduction mechanism. A,B) SECM lateral scans at constant height (20 µm). A) Traces and average (thicker lines) of the H_2_O_2_ oxidation currents measured at black platinum working electrode biased at 0.4 V versus Ag/AgCl (KCl 3 m). B) Box plots report maximal current amplitude, box limits report 1–99% frequency of the estimated distributions. PCPDTBT, *N* = 6; GLASS, *N* = 5; ***p < 0.001. C,D) Data representing the percentage of light‐ effect normalized over dark condition in hPSC‐CMs plated on PCPDTBT with or without catalase (CAT): Half time Ca^2+^ transient decay (*t*_1/2_, C) and spontaneous beating frequency (Frequency, D). PCPDTBT, *N* = 75, PCPDTBT+CAT, *N* = 75. *p < 0.05 with versus without CAT. Student's *t*‐test assay. E) Temperature variation in the proximity of the glass or PCPDTBT surface upon red light photoexcitation (power density, 20 mW mm^−2^). F) Variation (%) of *t*_1/2_ and spontaneous beating frequency obtained by increasing the bath temperature up to 40 °C through a heating chamber, normalized over physiological temperature (37°), observed in hPSC‐CMs plated on glass. *N* = 36.

Thus, we focused our attention on photoelectrochemical reactions, leading to the production of ROS at subtoxic concentration. As a first step, we verified that optical stimulation of PCPDTBT, without cells, leads to generation of ROS. To this goal, we employed the scanning electrochemical microscopy (SECM) technique (**Figure** **7**A,B), since it provides exquisite sensitivity to surface electrochemical processes in an aqueous environment. This technique has been applied to quantify the electrochemical activity at material/electrolyte interface and the metabolic activity of single cells and tridimensional cultures and tissues, with high spatial and temporal resolution.^[^ ^26^, ^72^, ^73^, ^74^, ^75^, ^76^, ^77^ ^]^ Spatially controlled illumination of conjugated polymer coupled with spatially resolved quantification of ROS production at the polymer/electrolyte interface has been recently reported for P3HT film.^[^ ^73^ ^]^

In the present work, we measured light‐induced ROS concentration in the solution in close proximity to the electrolyte/polymer interface, at ≈20 µm from the surface of PCPDTBT film, a distance of the same order of magnitude of cellular dimensions (Figure S6A, Supporting Information). The light spot size had a diameter of ≈100 µm (Figure S6B, Supporting Information). ROS were detected by employing platinum microelectrodes modified with black platinum as SECM probes, by applying an appropriate working potential (+0.4 V vs Ag/AgCl (KCl 3 m)). This modification has been showed to confer specificity and high sensibility toward ROS.^[^ ^72^, ^78^ ^]^ Considering that most of ROS species are characterized by very short lifetimes, and that they mainly end up in hydrogen peroxide (H_2_O_2_), by measuring H_2_O_2_ also the total amount of generated ROS is estimated. Figure 7A,B shows oxidation current signals recorded in lateral scans over illuminated regions of PCPDTBT films and glass substrates. The comparison between the two curves confirms the localized production of H_2_O_2_ following illumination of PCPDTBT thin films. The oxidation current resulting from H_2_O_2_ detection at the black platinum working microelectrode suggests that the local concentration of ROS produced at the PCPDTBT/water interface, at around 20 µm from the polymer surface, is ≈700 ± 200 nm. H_2_O_2_ concentrations have been obtained by converting oxidation currents with the titration curve for black platinum microelectrodes, as recently reported.^[^ ^72^ ^]^ Chronoamperometry measurements (Figure S7, Supporting Information), confirm that the current is generated by optical excitation of the polymer (the current is detected only in correspondence of optical excitation) and that both signal increase/decay dynamics occur over sub‐ms timescale.

In order to proof the interplay between optically generated ROS and modulation of Ca^2+^ dynamics triggered by PCPDTBT photoexcitation, we repeated Ca^2+^ measurements upon addition of a ROS quencher (Figure 7C,D). To this purpose we used catalase (CAT, 500 U mL^−1^), as a well‐known antioxidant enzyme which efficiently degrades H_2_O_2_.^[^ ^79^ ^]^ Interestingly, scavenging H_2_O_2_ production with CAT significantly reduced the combined effect of PCPDTBT and light on Ca^2+^ parameters. In particular, *t*_1/2_ acceleration was slightly reduced (−7% ± 1% PCPDTBT vs −6% ± 1% PCPDTBT+CAT, Figure 7C), while the beating frequency enhancement (about +70% in absence of CAT) was almost completely prevented in the CAT presence (about +14%. Figure 7D).

Overall, these observations support the conclusion that photoelectrochemical reactions occurring at the polymer/cell interface and leading to a H_2_O_2_ concentration increase play a significant role in the optical modulation of Ca^2+^ spontaneous beating by PCPDTBT.

Recently, it was demonstrated that blue and green monochromatic light stimulation leads to an increased H_2_O_2_ concentration, which in turn inhibits the mitochondrial activity and ultimately suppresses the Ca^2+^ transient and the contractility in CMs from mouse embryonic stem cell;^[^ ^1^ ^]^ however, no effect was detected in the case of exposure to red light. Likewise, a previous study from our group demonstrated that H_2_O_2_ plays a crucial role in the Ca^2+^ response to optical excitation of P3HT thin films in endothelial cells.^[^ ^22^ ^]^ In this study, Negri et al.^[^ ^22^ ^]^ showed that the production of ROS at the polymer/cell interface triggers the intracellular Ca^2+^ response through activation of the Transient Receptor Potential (TRP) Vanilloid 1 (TRPV1) channel, a member of the TRP channel family, previously shown to act as biosensor of cellular redox states^[^ ^80^ ^]^ and to be able to regulate the beating of CMs, through their permeability to Na^+^ and Ca^2+^_._^[^ ^81^ ^]^ Notably, it was recently reported that the TRP canonical isoform 7 (TRPC7) regulates the spontaneous Ca^2+^‐driven events in embryonic stem cell‐derived CMs through the modulation of the activity of RyR, SERCA, and NCX.^[^ ^82^ ^]^ Since our data demonstrated the involvement of SERCA and NCX in the polymer‐optical modulation, we hypothesize a role for TRP channels in the phototransduction mechanisms mediated by PCPDTBT. To further support our hypothesis, there is evidence that SERCA and NCX are direct targets of redox signaling.^[^ ^68^, ^69^ ^]^ Physiological redox regulation of SERCA can either increase or decrease its activity.^[^ ^69^ ^]^ On the one hand, ROS have been shown to inhibit SERCA function,^[^ ^83^ ^]^ as happens in some pathological conditions associated with increased oxidative stress;^[^ ^84^ ^]^ on the other hand, the redox regulation of phospholamban (PLN), a reversible regulator protein of SERCA, also results in its dissociation and thus in SERCA activation.^[^ ^85^ ^]^ Notably, evidence obtained in vascular smooth muscle cell indicates that a controlled oxidation of SERCA can increase its activity.^[^ ^86^ ^]^ In addition, Zhang et al. demonstrated that red LED light irradiation promotes ATP synthesis, speculating that red LED may modulate the activity of myocardial proteins, including SERCA, in mice CMs.^[^ ^53^ ^]^ Since our findings indicate an increase of SERCA activity by PCPDTBT‐mediated optical stimulation, we can hypothesize that this effect is due either to i) a direct and controlled modulation of SERCA or to ii) an indirect modulation through PLN.

NCX can also be directly activated by ROS;^[^ ^83^ ^]^ in particular, the activation of NCX by high concentration of ROS has been shown to result in a Ca^2+^ overload under pathological condition such as ischemia‐reperfusion;^[^ ^87^ ^]^ conversely, the exposure of adult rat CMs to modest (100 µm) concentration of H_2_O_2_ does non induce intracellular Ca^2+^ accumulation through NCX activation.^[^ ^83^ ^]^ Our findings show that during NCX inhibition, the optical stimulation of PCPDTBT accelerates the intracellular Ca^2+^ decay; moreover, in the absence of blockers, the intracellular Ca^2+^ accumulation observed in GLASS (+12.5%) is not present in PCPDTBT group (Figure S8, Supporting Information). These findings are consistent with an indirect modulation of NCX by PCPDTBT‐induced ROS production under illumination.

In addition to the mechanisms described above, modulation of cardiac voltage‐gated ion channels may also be involved, as a consequence of ROS increase.^[^ ^88^ ^]^ For instance, oxidation of Na^+^ and L‐type Ca^2+^ channels has been associated to AP prolongation and pro‐arrhythmogenic events, such as early after depolarization (EAD).^[^ ^89^ ^]^ However, illumination of PCPDTBT led to APD shortening and no arrhythmic events were observed in our experiments, allowing to discard this effect as a relevant pathway. Conversely, our findings are in line with the activation of ATP sensitive K^+^ channels by ROS‐induced ROS release from mitochondria,^[^ ^90^ ^]^ which leads to shortened APD.

The experimental evidence and literature reports discussed above are not per se sufficient to completely disregard a possible contribution photothermal effect, which have been also reported to actively mediate modulation of cell behavior and tissue functionality by organic semiconductors.^[^ ^16^, ^91^ ^]^

In fact, the photothermal stimulation may potentially play a role concomitant to ROS production, based on the following considerations: i) H_2_O_2_ inhibition did not completely revert the PCPDTBT‐induced photoexcitation effect on CaT decay; so an additional process might be at play; ii) the link between photo‐ and thermal effect on the modulation of iPSC‐CMs seeded on a conjugated polymer has been shown recently;^[^ ^25^ ^]^ the authors ascribed the photo‐induced contraction response to the photothermal effect through the activation of the Ca^2+^ handling proteins, such as SERCA; and iii) it is known that SERCA's activity is temperature‐dependent.^[^ ^92^ ^]^ All studies shown here were performed at 37 °C, but polymer optical excitation may lead to a localized temperature increase above physiological temperature. Importantly, the cellular functionality modifications induced by near infrared/infrared induced temperature enhancement^[^ ^93^ ^]^ and the TRPV channels activation by temperature changes are well described;^[^ ^94^ ^]^ iv) TRPV channels activation has been related to intracellular Ca^2+^ handling variation (TRPV4)^[^ ^95^, ^96^ ^]^ and beating regulation (TRPV1).^[^ ^97^ ^]^ Based on these considerations, we cannot disregard the involvement of photothermal effect in the Ca^2+^ homeostasis modulation by optical stimulation of PCPDTBT. In order to address this hypothesis, we first directly estimate the local heating due to red photoexcitation (power density within the range 0–20 mW mm^−2^), in the proximity of the polymer/extracellular bath interface, through the calibrated pipette resistance method.^[^ ^98^ ^]^ The maximum temperature increase amounts at 3.3 ± 0.2 °C (equilibrium temperature around 40 °C) in the proximity of the PCPDTBT surface upon exposure to the highest light density; instead, no heating at the GLASS surface was detected (Figure 7E). The temperature profile reached a steady state plateau value about 500 ms after illumination onset. No cell damage however was observed (data not shown). At power densities lower than 5 mW mm^−2^, the temperature increase is negligible (Figure S9, Supporting Information).

Data reported in Figure 7E allow to disregard any direct effect of water environment heating in absence of the polymer; however, it is necessary to verify whether the temperature increase recorded in the extracellular bath in the presence of the polymer has an impact, by itself, on observed modulation effects. To this goal, we evaluated the variation in CaT decay and frequency by increasing the temperature up to 40 °C in absence of polymer and light, by using a heating chamber. Increment of temperature to 40 °C enhanced both the CaT decay and the spontaneous beating frequency (Figure 7F). However, the effect on CaT decay time is opposite to the one induced by PCPDTBT photoexcitation, possibly due to a different kinetics in heat dissipation processes associated to the two heating methods. On the other hand, when the temperature was raised to 40 °C in the bath, the spontaneous beating frequency increased by 10% (Figure 7F left), which is the same percentage increase induced by the photoexcitation of PCPDTBT in the presence of ROS quencher (Figure 7D).

Taken together these results support the hypothesis that both photothermal and photoelectrochemical processes mediated by polymer photoexcitation play a role in the modulation of Ca^2+^ homeostasis. In more detail, ROS generation primarily (roughly 90% of the effect) affects the beating frequency, while localized heat generation following polymer illumination determines the decrease in the decay time.

### 2.6 Perspective Therapeutic Applications

**Figure 8:**
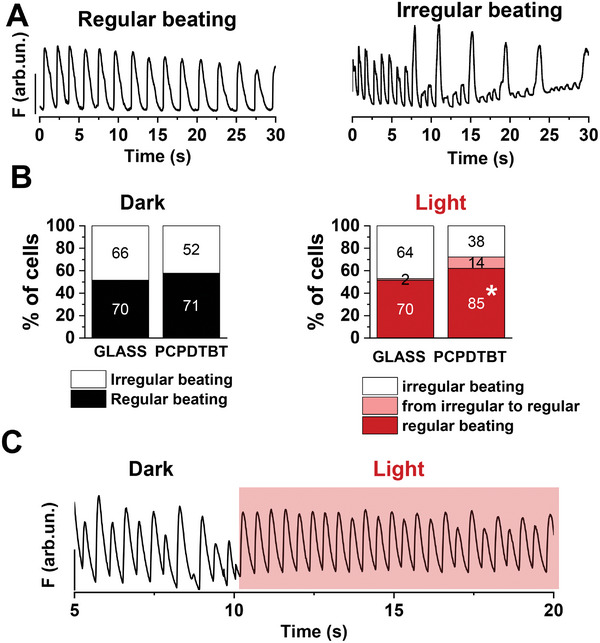
Antiarrhythmogenic effect of PCPDTBT optical stimulation. A) Representative regular (left) and irregular (right) profiles of spontaneous Ca^2+^ activity. B) Qualitative analysis of spontaneous beating patterns: regular beating, irregular beating and cells which switch from the irregular to the regular beating. C) Example of Ca^2+^ dynamics in dark condition and under illumination. GLASS, *N* = 136 and PCPDTBT, *N* = 123. *p < 0.05 GLASS versus PCPDTBT (Chi‐square test).

Looking ahead of clinical application, it is important to evaluate whether the excess amount of ROS can induce electrical instability and cardiac arrhythmogenic events, as reported in some literature reports.^[^ ^69^, ^99^ ^]^ We thus investigated whether the PCPDTBT‐photoexcitation influences the spontaneous beating pattern of hPSC‐CMs, resulting in arrhythmia. To this end, we first performed a qualitative analysis of spontaneous beating patterns: hPSC‐CMs were divided into two different groups based on their Ca^2+^ dynamics profile: regular or irregular (**Figure** **8**A). hPSC‐CMs exhibiting arrhythmogenic characteristics, such as spontaneous Ca^2+^ release or heterogeneous of CaT,^[^ ^100^ ^]^ were classified as irregular beating pattern. In the dark condition, percentage of regular and irregular spontaneous beating patterns were comparable between PCPDTBT and GLASS groups (Figure 8B, left); on the contrary, upon illumination, the proportion of hPSC‐CMs exhibiting a regular beating pattern was significantly higher on PCPDTBT compared to cells on GLASS (Figure 8B, right). Surprisingly, 26.9% (14/52) of the CMs showing an irregular beating switched to a regular beating pattern in PCPDTBT under optical stimulation; whereas the switch was present only in the 3% (2/66) of the cells plated on GLASS, sustaining a role of PCPDTBT photomodulation in normalizing beating activity of hPSC‐CMs. Representative Ca^2+^ dynamics traces before and during illumination are shown in Figure 8C. The use of light (photobiomodulation) has been proposed as alternative therapeutic approach of heart failure,^[^ ^53^ ^]^ ischemia/reperfusion damage^[^ ^101^, ^102^ ^]^ and cardiac arrhythmias,^[^ ^103^ ^]^ although in the latter case genetic manipulation was involved (optogenetics). Nyns et al.^[^ ^103^, ^104^ ^]^ showed that ventricular arrhythmias and atrial fibrillation are ceased after illumination in optogenetically modified adult rat heart with light‐gated depolarizing ion channel red‐activatable channelrhodopsin (ReaChR). Our data strongly suggest a possible antiarrhythmic effect triggered on demand by PCPDTBT photoexcitation; thus, they overall support the potential use of conjugated polymer mediated optical stimulation as a new, geneless therapeutic approach to cardiac electric abnormalities.

We can envision clinical application of our approach to inherited arrhythmias or for the treatment of the consequences of HF. An example of a cardiac pathology characterized by occurrence of deadly arrhythmic events is Long QT Syndrome (LQTS). LQTS is a cardiac disorder where patients exhibit a prolongation of the QT interval when examined through an electrocardiogram,^[^ ^105^ ^]^ which reflects an extended APD and CaT duration^[^ ^106^ ^]^ at cellular level. Individuals with LQTS are more susceptible to episodes of *torsades de pointes*, a specific type of ventricular tachycardia, which can lead to sudden cardiac arrest, if not promptly treated.^[^ ^105^ ^]^ This work shows that the photoexcitation of PCPDTBT, in addition to the antiarrhythmic effect, reduces APD and increases the CaT decay (Figures 3 and 4), thus it may potentially reduce QT prolonged intervals. Therefore, photomodulation could offer a promising therapeutic avenue for some of the most severe LQTS patients, currently at risk of developing potentially life‐threatening arrhythmias.

In addition, our findings might have significant implications also for HF, the common endpoint of various cardiovascular disorders and occurring in most acute myocardial ischemia patients. HF is characterized by the heart's inability to maintain the blood pumping function.^[^ ^107^ ^]^ Studies in both humans and animals widely demonstrated that SERCA activity is reduced in HF,^[^ ^108^ ^]^ deteriorating cardiac performance and further accentuating the symptoms of HF. Owing to these evidences, the enhancement of SERCA activity has emerged as a therapeutic target in HF treatments.^[^ ^108^ ^]^ Our study demonstrates that the light stimulation of polymer enhances SERCA activity. Thus, light and conjugated polymers in synergy may provide a groundbreaking alternative in HF treatment, overcoming some of the limitations typically associated with conventional drug therapies.

Based on our discoveries, the innovative use of photoexcitation of conjugated polymer offers new tools for the treatment of cardiovascular disease. The transition from bench to bedside will be hopefully fastened not only by the peculiar features typical of conjugated polymers, such as the possibility to realize highly conformable and fully biocompatible, implantable devices, but also by the availability of commercial, implantable optical fibers, capable to provide highly directional illumination at the required wavelength and power density.

As the next necessary step, safety, efficacy, and long‐term reliability of the proposed approach will be addressed in vivo, in pathologically relevant models, and will certainly foster exciting clinical research.

## 3 Conclusion

In this study, we investigated the feasibility of photomodulation mediated by a red‐absorbing conjugated polymer (PCPDTBT) in human CMs, deepening its effects on functional properties of hPSC‐CMs. The main findings of the present study showed that, under photoexcitation, PCPDTBT efficiently influences the hPSC‐CMs phenotypic features by i) modulating the membrane properties (APD shortening); ii) increasing the beating frequency; iii) accelerating the Ca^2+^ decay through an indirect modulation of SERCA and NCX. In addition, we found out that the optical stimulation of PCPDTBT is able to exert an antiarrhythmogenic effect on spontaneously beating hPSC‐CMs.

**Figure 9:**
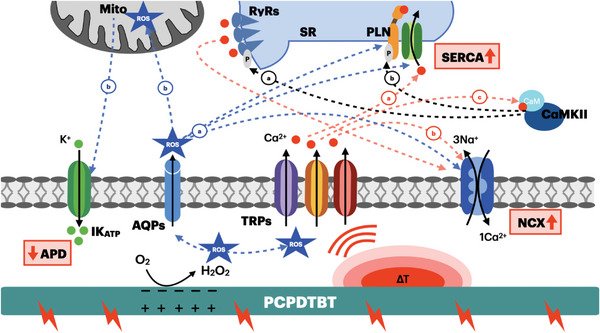
Hypothesized biophysical pathways triggered by PCPDTBT photoactivation. Mito: Mitochondrion, RYR: ryanodine receptors; SR: Sarcoplasmic Reticulum; PLN: Phospholamban; SERCA: Sarco‐Endoplasmic Reticulum Calcium ATPase; CaM: Calmodulin; CaMKII: Calmodulin‐Dependent Protein Kinase II; IK_ATP_: ATP‐sensitive K^+^ current; AQPs: aquaporins; TRP: Transient receptor potential channels; NCX: Na^+^/Ca^2+^ exchanger; ROS: Reactive Oxygen Species; H_2_O_2_: Hydrogen peroxide.

We also demonstrated that PCPDTBT photoexcitation supports mainly two phototransduction phenomena in hPSC‐CMs: the photoelectrochemical ROS production, which leads to beating frequency increment, and the local temperature enhancement, which induces a faster intracellular Ca^2+^ decay. **Figure** **9** shows the hypothesized biophysical pathways triggered by these two phenomena. Optical stimulation of PCPDTBT results in a ROS production and temperature enhancement, which could directly activate the TRP channels family. Ca^2+^ permeated through TRP channels is expected to modulate Ca^2+^ dynamics, by accelerating, directly or indirectly, the removal of cytosolic Ca^2+^. This results in the observed decrease of Ca^2+^ decay time. Intracellular Ca^2+^ could directly influence both SERCA (red arrow, (a)) and NCX activities (red arrow, (b)), promoting the movement of Ca^2+^ from the cytosol into the SR via SERCA or out of the cell by NCX. On the other hand, Ca^2+^ could activate the complex of CaM‐CaMKII (red arrow (c)) which in turn could phosphorylate i) PLN, consequently increasing SERCA activity (black arrow (a)) and ii) RyRs on the SR (black arrow (b)), resulting in localized, additional Ca^2+^ release. The ROS produced by optical stimulation of PCPDTBT in the space between the polymer and the cell membrane can also cross the cell membrane directly through aquaporins. Thereby, ROS may directly modulate the activity of proteins involved in Ca^2+^ homeostasis (SERCA, PLN, and NCX, blue arrows (a)). In addition, they may also modulate ATP sensitive K^+^ channels activation by ROS‐induced ROS release from mitochondria, resulting in an outward K^+^ current and, consequently, a reduction of action potential duration (APD, blue arrows (b)). Taken together, these modulation mechanisms may explain the observed faster Ca^2+^ dynamics, increase in spontaneous beating frequency and the antiarrhythmogenic effect triggered by the polymer.

Altogether, our results provide compelling evidence that the use of an efficient red‐absorbing conjugated polymer represents an opportunity for finely tuned control of CMs functionality.

Although further studies are needed to exploit the full potential of this technology in clinically relevant applications, this work is a fully original proof of concept that conjugated polymers and red light excitation, in synergy, hold the potential to treat several cardiac diseases, as a fully unexplored therapeutic approach.

## 4 Experimental Section

### Conjugated Polymer Thin Films Preparation and Characterization

PCPDTBT powder was purchased from 1‐Material and used as received (*M*_W_, 40 000–50 000). PCPDTBT solution in chlorobenzene (concentration, 20 mg mL^−1^) was stirred for 20 h and heated at 60 °C prior deposition. Glass substrates were carefully cleaned by subsequent rinses (10 min in ultrasonic bath) in deionized water, acetone, and isopropanol, respectively. Polymer thin films were fabricated by spin‐coating technique, with one single spin coating step (1500 rpm, acceleration 1500 rpm s^−1^, 60 s), obtaining homogeneous coverage of the glass substrate with an average film thickness of about 150 nm (measured by stylus profilometry). Thermal sterilization (2 h, 120 °C) completed the fabrication process. The UV–vis optical absorption spectrum of the PCPDTBT thin film was acquired with a Perkin–Elmer Lambda 1050.

### Human Pluripotent Stem Cells Maintenance and Cardiac Differentiation

RUES2 hPSCs were cultured onto RH‐vitronectin (ThermoFisher Scientific) coated dishes in StemFlex medium (ThermoFisher Scientific) and passaged when 80–90% confluence was reached, using 0.5 mm EDTA after pre‐treatment with 5 µm ROCK inhibitor—Y27632 (Selleckchem). Differentiation into cardiomyocytes (CMs) was performed using a chemically‐defined serum‐free protocol based on the modulation of the Wnt pathway in RPMI medium supplemented with B‐27 without insulin.^[^ ^109^, ^110^ ^]^ Specifically, the protocol entails the treatment of 90–95% confluent hPSCs with the GSK3β inhibitor CHIR99021 (Selleckchem) for 24 h, resulting in the activation of the Wnt pathway, followed by its inhibition by IWR‐1 treatment at day 3, for 48 h. Contracting cells usually appear in a range between d7‐d10 of differentiation. At d10, insulin is added to the medium. At d14, T3 hormone (triiodothyronine), dexamethasone (DEX), and IGF‐1 (insulin‐like growth factor‐1) are also added to improve maturity of the cells.^[^ ^111^ ^]^

hPSC‐CMs were then used for the experiments around d20‐d25 after beating starts. For the experiments, cells were plated onto either glass or polymer, both coated with fibronectin (5 µg cm^−2^) and laminin (5 µg cm^−2^), at about 50–70% confluency, and analyzed 2–3 days after seeding.

All experiments were performed at physiological temperature (37 °C) using a volume‐controlling chamber (Warner Instruments).

### Ca^2+^ Imaging Experiments

Ca^2+^ dynamics were recorded in spontaneously beating hPSC‐CMs incubated with 2.5 µm Fluo4‐AM (Life Technologies) for 20 min in extracellular solution, prepared as it follows (nm): 137 NaCl, 5.4 KCl, 2 CaCl_2_, 1 MgCl_2_, 10 Hepes, 10 Glucose, pH adjusted to 7.4 with NaOH. After Fluo4‐AM incubation, cells were washed out for 10 min with fresh extracellular solution before recordings. Ca^2+^ imaging experiments were performed on Fluo4‐AM loaded‐cells plated on polymer or on glass control samples, by using an upright microscope (Olympus BX63) equipped with a 40x objective and a sCMOS Camera (Prime BSI, Teledyne Photometrics; Tucson, AZ, USA). Fluo4‐AM photoexcitation was provided by a LED light source (Lumencor Spectra III, emission maximum at 474 nm, 5.8 mW mm^−2^) fiber‐coupled to the microscope. Fluo4‐AM has a negligible spectral overlap with the PCPDTBT optical absorption (Figure S1A, Supporting Information). During Fluo4‐AM fluorescence acquisition, PCPDTBT films were simultaneously excited for 30 s by using a LED light source (Lumencor SPECTRA III, maximum emission peak at 660 nm, band FWHM about 50 nm) or a laser (Lumencor CELESTA, emission peak centered at 660 nm, band FWHM about 10 nm), whose emission was coupled to the optical path of the microscope. The use of laser excitation was preferred in all cases where a higher excitation density was preferable (namely, 20 mW mm^−2^). Light power density was varied within the range 6–20 mW mm^−2^. Photoexcitation density was measured in correspondence to the light spot size, at the same focal plane of the cell culturing substrate, in all cases.

Variation of fluorescence signal intensity was assessed over Region of Interest (ROI) covering single cells area. Image processing and data analysis were carried out with ImageJ software and Origin2018, respectively.

Partial SERCA blockade was obtained by incubation of hPSC‐CMs with 10 µm thapsigargin (Sigma‐Aldrich) for 15 min and washed out. Pharmacological inhibition of NCX) and extracellular ROS) was carried out by administration of 2 µm SEA0400 (Sigma‐Aldrich) and 500 µ mL^−1^ CATALASE (Sigma‐Aldrich), respectively, dissolved in extracellular solution.

### Electrical Activity

Electrophysiology recordings were performed by using a Axopatch 400 amplifier (Axon Instruments) coupled with the same microscope employed for Ca^2+^ imaging experiments. Action potentials (AP) were measured by employing the perforated patch‐clamp technique (I‐clamp mode) using pore‐forming antibiotic amphotericin B (0.22 mm) at physiological temperature (37 °C). The intracellular solution contained (in mm): 110 K‐aspartate, 23 KCl, 3 MgCl_2_, 0.04 CaCl_2_, 0.1 EGTA KOH (10^−7^ Ca^2+^‐free), 5 Hepes KOH, 0.4 Na^+^‐GTP, 5 Na^+^‐ATP, 5 Na^+^‐phosphocreatine, pH adjusted to 7.3 with KOH. The extracellular solution consisted in (mm): 137 NaCl, 5.4 KCl, 2 CaCl_2_, 1 MgCl_2_, 10 Hepes, 10 Glucose, pH adjusted to 7.4 with NaOH. In some cases, the cells were paced with an electrical stimulation at 1 Hz. PCPDTBT films were excited for 30 s through a laser (Lumencor CELESTA, emission peak centered at 660 nm, band FWHM about 10 nm, 20 mW mm^−2^), fiber‐coupled to the optical path of the microscope.

Data were acquired with the pCLAMP10 software (Axon Instruments) and then analyzed with Clampfit (Axon Instruments) and Origin 8.0 (OriginLab Corporation).

### MTT Assay

Methylthiazolyldiphenyl‐tetrazolium bromide (MTT) (Sigma‐Aldrich) assay was performed to assess cell viability. hPSC‐CMs cultured onto either glass control or polymer samples were incubated with 1 mg mL^−1^ MTT, dissolved in RPMI medium, for 4 h at 37 °C. After rinsing with PBS, 200 µl per well of solubilization solution made by dimethyl sulfoxide (DMSO) were added and incubated for 1 h at 37 °C. Samples were then transferred to a 96‐wells plate and the absorbance was measured at 570 nm.

### Scanning Electrochemical Microscopy (SECM)

Scanning electrochemical microscopy measurements were performed with a CHI910B SECM instrument from CH Instruments Inc. (Austin, Texas). A three‐electrodes setup was used, by employing Ag/AgCl (KCl 3 m) as the reference electrode and a platinum wire as the counter electrode. The employed working electrodes for ROS detection were platinum microelectrodes with a 10 µm active disk (CHI Pt 10 µm, RG 10), modified by black platinum electrodeposition, as previously reported.^[^ ^72^, ^112^ ^]^ PCPDTBT thin films immersed in Hank's Balanced Salt Solution (HBSS) were illuminated with a light spot of ≈100 µm diameter (Figure S6B, Supporting Information); photostimulation was obtained through a mercury lamp of a Nikon Eclipse Ti inverted microscope, filtered with a Nikon Texas Red HYQ cubic filter (excitation wavelength, 532–587 nm; emission wavelength, 608–683 nm). The power density of the photoexcitation source was ≈20 mW mm^−2^. Lateral SECM scans over the illuminated region at a height of about 20 µm from the PCPDTBT surface were performed by biasing the working electrode (the probe) at 0.4 V versus Ag/AgCl (KCl 3 m). The height of the SECM scan was defined based on SECM probe scan curve on the normal direction to the PCPDTBT film (*z* axis in Figure S6A, Supporting Information) and measuring oxygen reduction currents at −0.6 V versus Ag/AgCl (KCl 3 m). Chronoamperometric measurements of ROS oxidation were recorded with black platinum working electrodes positioned at the center of the light spot at a distance of 20 µm from the PCPDTBT surface.

### Evaluation of Photothermal Effect

Calibrate pipette resistance method^[^ ^98^ ^]^ was used to measure the light‐induced temperature variation within the extracellular solution in the chamber, in the close proximity (about 1 µm) to the polymer or control glass surface. The measurements were carried out upon the same photoexcitation conditions adopted in the whole work and by employing the same electrophysiology setup. The sample was exposed to an extracellular solution of the same composition employed for cell electrophysiological recordings. The glass micropipette electrode was filled with the same medium present in the bath, in order not to have liquid junction potentials arising at the pipette tip. The measurements were performed in voltage clamp configuration by applying a constant current of *I*_0_ = 4 nA, at fixed basal temperature of 37 °C. The light‐induced measured current is related to the temperature variation through the following relation:

(1) $$T=\frac{1}{\frac{1}{T_{0}}-\frac{R}{E_{a}}\;\log\left(\frac{I}{I_{0}}\right)}$$

where *I*_0_ is the current flowing through the pipette at the basal temperature *T*_0_, *R* is the ideal gas constant, and *E*_a_ is the activation energy. *E*_a_ was obtained by measuring the current response flowing through the pipette for a potential step of Δ*V* = 5 mV at different values of the bath temperature, controlled with a volume‐controlling chamber (Warner Instruments), and plotting the measured currents against the temperature in an Arrhenius plot.

### Statistical Analysis

The significance of differences was assessed with unpaired/paired Student's *t*‐test for continuous variables (Ca^2+^ dynamics and Electrophysiology parameters). Chi‐square test was applied to compare the categorical variables (number of cells showing Ca^2+^ events). Continuous variables are represented as means ± standard errors, while the categorical variables are expressed in percentages. Statistical significance was defined as *p* < 0.05. Sample size (*n*, number of cells) is specified for each experimental condition in the figure legends. Statistical analysis was achieved using Origin8.0 (OriginLab Corporation).

## Conflict of Interest

The authors declare no conflict of interest.

## Author Contributions

E.D.P. and M.R.A. contributed equally to this work. C.R.: Conceptualization, Data curation, Formal analysis, Investigation, Writing—original draft.; C.G.: Data curation, Investigation, Formal analysis.; G.T. and M.M.: Data curation, Investigation, Formal analysis, Writing—original draft; C.M.: Preparation of polymer thin films; C.G., M.M., and S.C: cell culture preparation; S.R. and E.D.P.: Conceptualization, Supervision, Writing—review & editing, and Funding acquisition; M.R.A.: Conceptualization, Supervision, Project administration, Writing—Original Draft, Writing—review & editing and Funding acquisition. All authors have given approval to the final version of the manuscript.

## Supporting information

Supporting Information

## Data Availability

The data that support the findings of this study are available from the corresponding author upon reasonable request.
