# Supplementary material for: Nongenetic Optical Modulation of Pluripotent Stem Cells Derived Cardiomyocytes Function in the Red Spectral Range
Source: Adv Sci (Weinh). 2023 Nov 10;11(3):2304303. doi: 10.1002/advs.202304303 (PMC10797444; doi:10.1002/advs.202304303)
Supplement: Supplementary file 1 — Supporting Information [file ADVS-11-2304303-s001.pdf]

ADVANCED SCIENCE
Open Access

Supporting Information

for *Adv. Sci.*, DOI 10.1002/advs.202304303

Nongenetic Optical Modulation of Pluripotent Stem Cells Derived Cardiomyocytes Function in the Red Spectral Range

Carlotta Ronchi, Camilla Galli, Gabriele Tullii, Camilla Marzuoli, Marta Mazzola, Marco Malferrari, Silvia Crasto, Stefania Rapino, Elisa Di Pasquale and Maria Rosa Antognazza**

SUPPLEMENTARY INFORMATION

Non-genetic optical modulation of pluripotent stem cells derived cardiomyocytes function in the red spectral range

C. Ronchi[1,], C. Galli[2], G. Tullii[1], C. Marzuoli[1,3], M. Mazzola[2], M. Malferrari[4], S. Crasto[2], S. Rapino[4], E. Di Pasquale[2,5,#], M.R. Antognazza[1, *,#]*

[1]Center for Nano Science and Technology@Polimi, Istituto Italiano di Tecnologia, 20133, Milano, Italy
[2] Humanitas Clinical and Research Center-IRCCS, 20089, Milan, Italy
[3]Politecnico di Milano, Physics Dept., P.zza L. Da Vinci 32, 20133 Milano, Italy
[4]University of Bologna, Department of Chemistry ''Giacomo Ciamician'', via Francesco Selmi 2, 40126 Bologna, Italy

[5]Institute of Genetic and Biomedical Research (IRGB), UOS of Milan – National Research Council of Italy (CNR), Milan, Italy

**corresponding authors*

# co-last authors

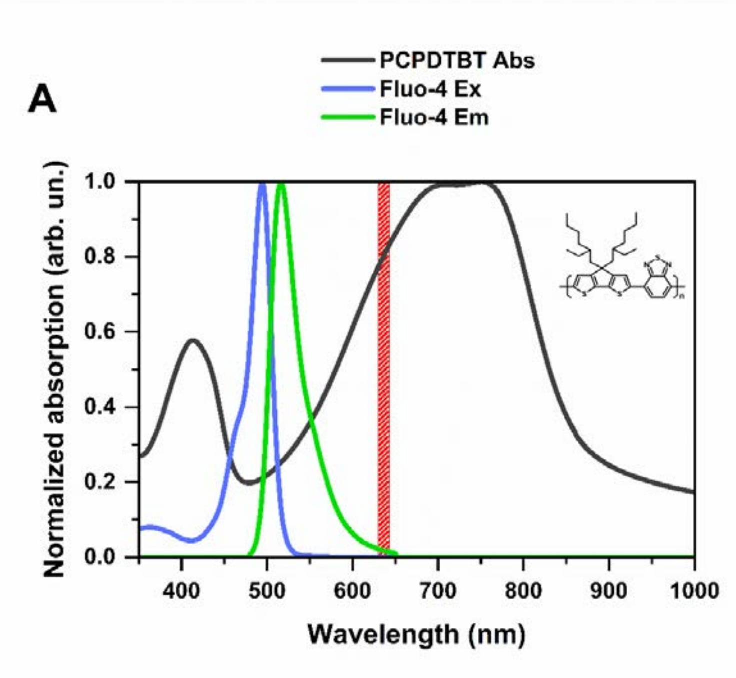

Figure S1 Optical absorption spectrum of PCPDTBT (black line) and Excitation (cyan line) and Emission spectra (green line) of Fluo4-AM. Inset shows the chemical structure of PCPDTBT.

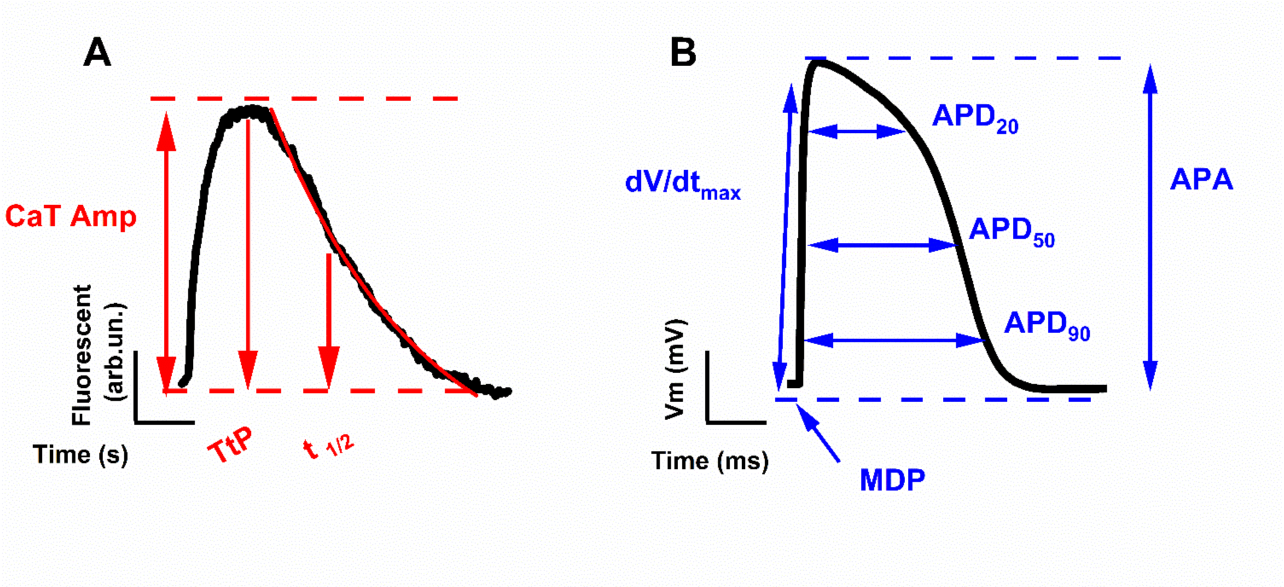

Figure S2. Ca^{2+} transient (left) and Action Potential (right) parameters. Ca^{2+} transient (CaT), peak amplitude (CaT Amp), defined as the difference between the fluorescence peak value and the baseline value, or ΔF/F; time to peak (TtP), defined as the time difference between the CaT onset and the CaT maximum; half-time CaT decay ($t_{1/2}$), defined as the half-time decay of CaT dynamics; maximum diastolic potential (MDP), defined as the resting membrane potential (V_m); AP amplitude (APA), defined as the potential difference between the AP peak and the MDP value; maximum upstroke velocity (dV/dt_{max}), defined as the first derivative of the AP depolarization phase; AP duration at fixed time points during the repolarization phase (APD90, APD50, APD20), defined as the time difference between the AP onset and the timing at which the AP value has decreased by 90%, 50%, 20%, respectively.

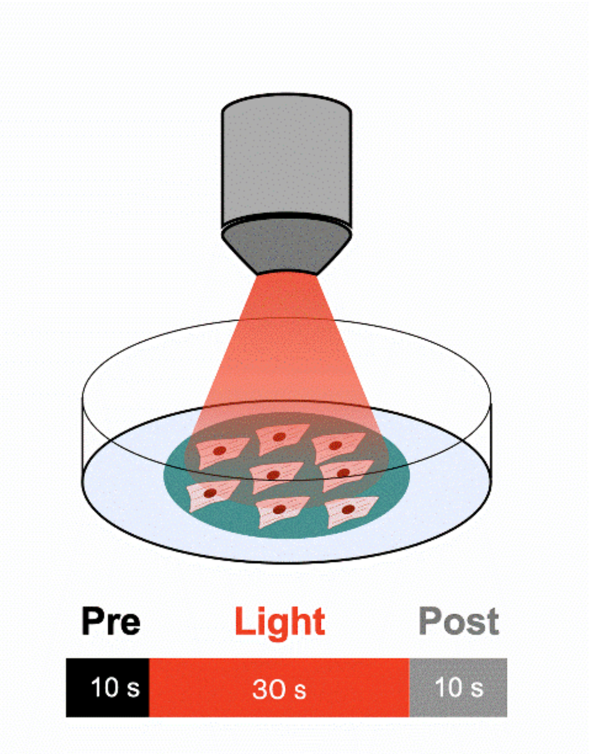

Figure S3. Schematic representation of the experimental set-up and protocol used for optical excitation.

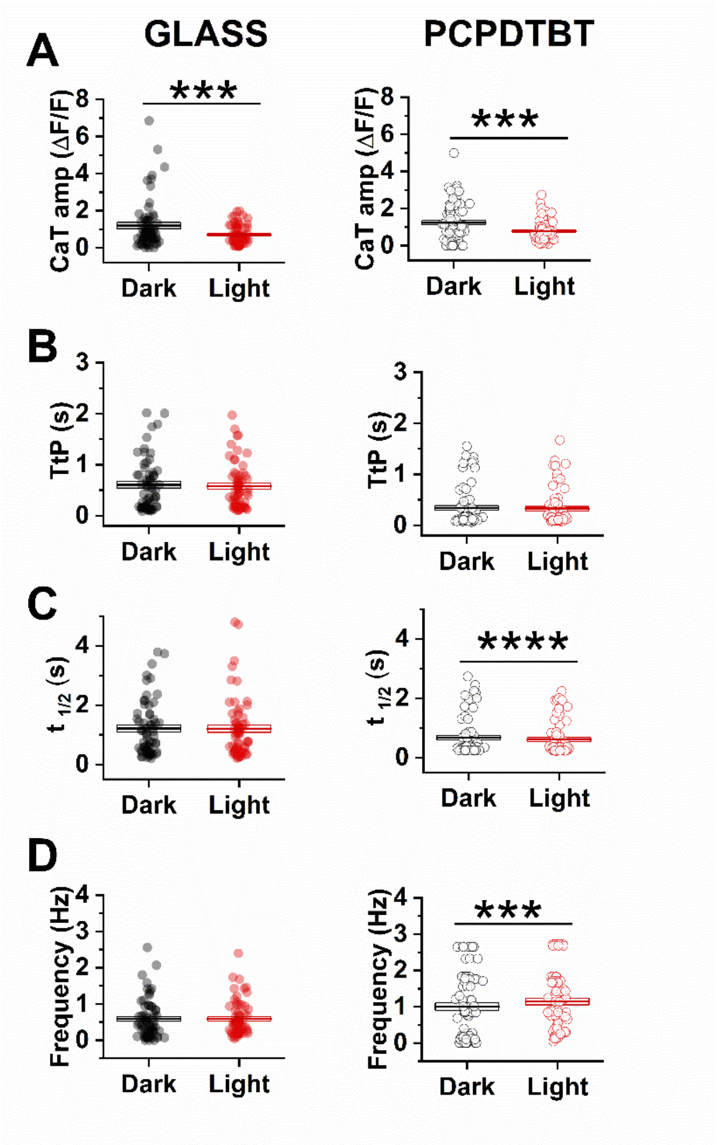

Figure S4. **Modulation of Ca^{2+} dynamics** in hPSC-CMs plated on GLASS (left) and PCPDTBT (right) in dark condition and under red-light photostimulation at 20 mW/mm^2. **A**) Ca^{2+} transient amplitude (CaT); **B)**Time to Peak (TtP); **C)** half time decay ($t_{1/2}$) and **E**) spontaneous beating frequency (Frequency). Individual and average ± SE (box) values are shown in each dot plot. GLASS and PCPDTBT N > 50. ° p < 0.05, *** p<0.001, **** p<0.0001 Dark vs Light (Paired Student's t -test)

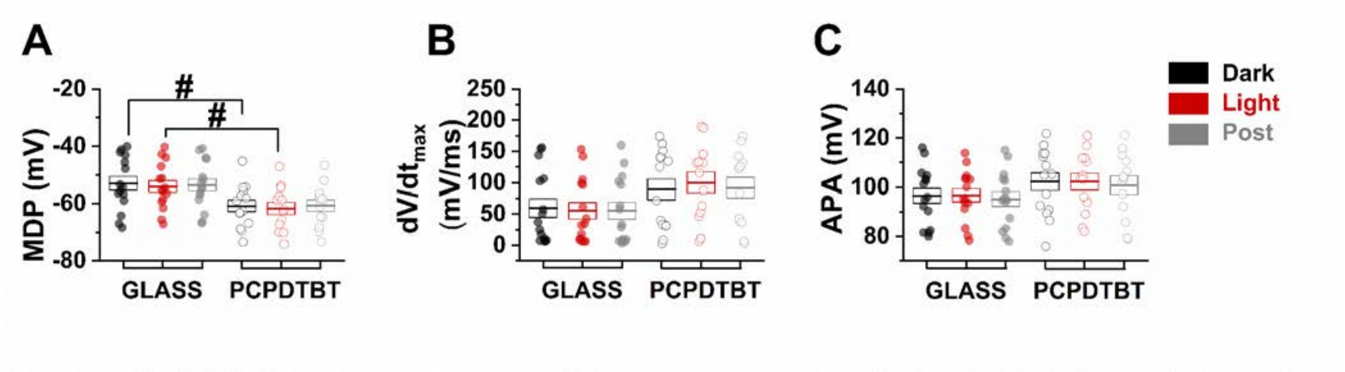

Figure S5. Optical stimulation of electrical activity of iPSC-CMs. Action Potential parameters evaluated before (Dark), during (Light) and after (Post) illumination during a constant rate pacing at 1Hz (**A)** maximal diastolic potential (MDP); **B)** maximal upstroke velocity (dV/dt_{max}); **C)** AP amplitude (APA). Individual and average ± SD (box) values are shown in each dot plot. GLASS N>10, PCPDTBT N >10, # $p < 0.05$ GLASS vs PCPDTBT (Student's t-test).

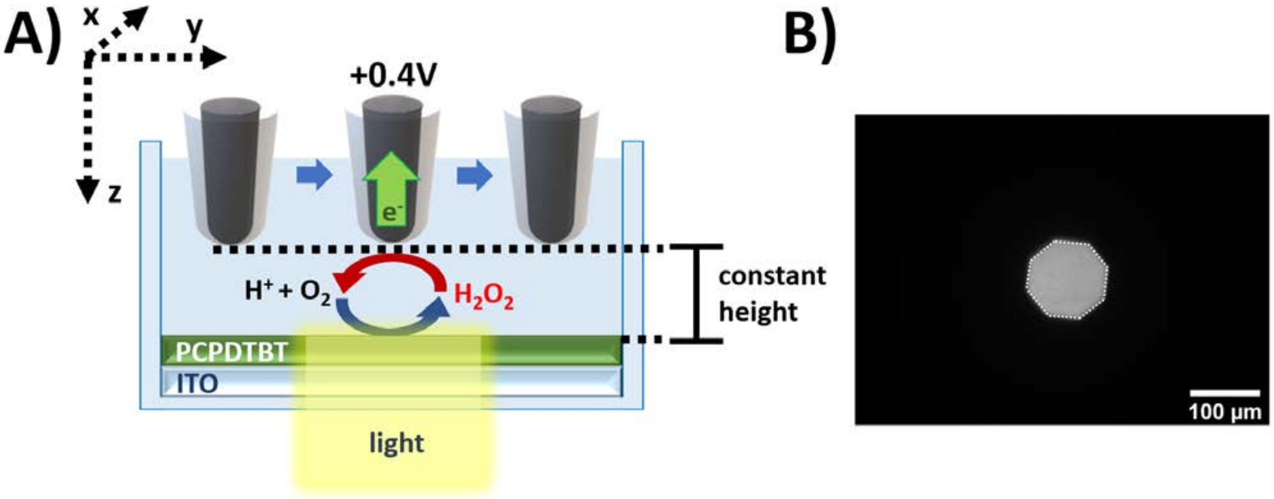

Figure S6. A) Schematic representation of SECM lateral scan at constant height over PCPDTBT surface. As detailed in the Materials and Methods section, the black platinum modified platinum microelectrodes were biased at +0.4 V vs Ag/AgCl (KCl 3 M) and moved along y direction at constant height, approximately 20 µm, from the PCPDTBT thin film. **B**) Image of the light spot size.

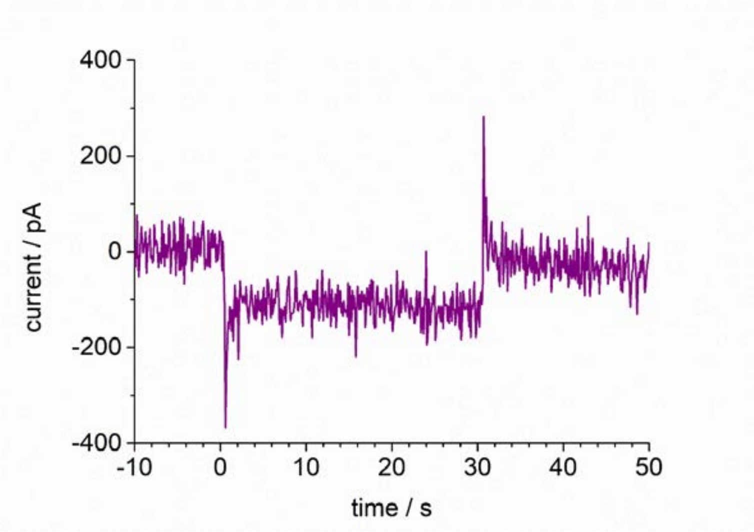

Figure S7. Time course of photoinduced ROS production, as measured by chronoamperometric detection of ROS. Light on, time = 0 s; light off, time = 30 s; wavelength, 532-587 nm; power density, 20 mW/mm^2

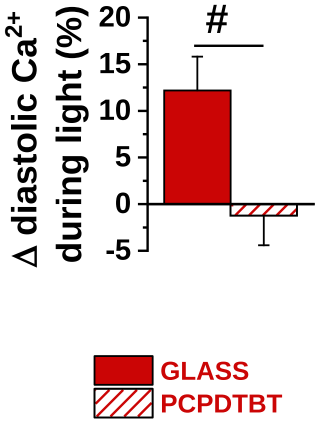

Figure S8. Effect of photostimulation of diastolic Ca^{2+} in hPSC-CMs plated on GLASS and on PCPDTBT. Percentage of relative variation vs dark condition during the light stimulation of diastolic Ca^{2+}. GLASS N = 55, PCPDTBT N= 73. # $p < 0.05$ GLASS vs PCPDTBT.

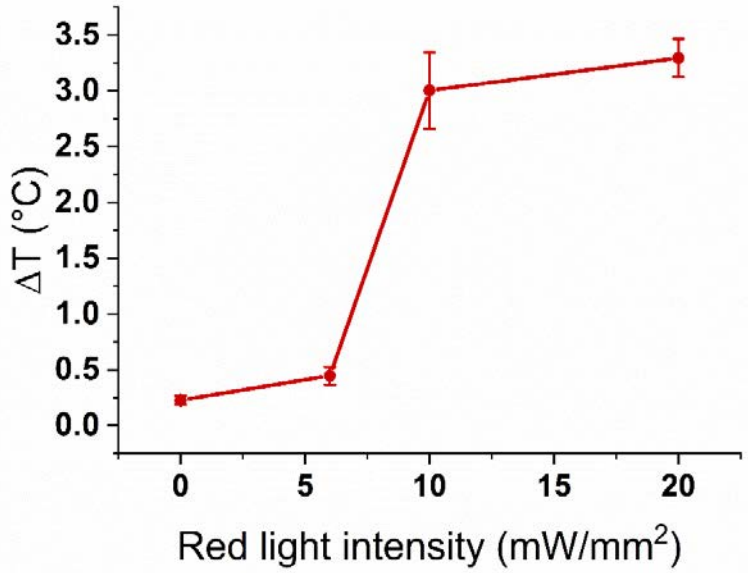

Figure S9. Temperature variation recorded in the extracellular solution in the close proximity of the PCPDTBT polymer, by employing the calibrated pipette resistance method, in dependence on red light power density. The polymer was exposed to the light for 30 seconds.
